# Supplementary material for: Polytypes and planar defects revealed in the purine base xanthine using multi-dimensional electron diffraction
Source: Commun Chem. 2025 Nov 5;8:331. doi: 10.1038/s42004-025-01729-2 (PMC12589599; doi:10.1038/s42004-025-01729-2)
Supplement: Supplementary file 2 — Supplementary Information [file 42004_2025_1729_MOESM2_ESM.pdf]

Supplementary Information

Polytypes and Planar Defects Revealed in the Purine Base Xanthine using Multi-Dimensional Electron Diffraction

Helen W. Leung[a]*, Royston C. B. Copley[b], Giulio I. Lampronti[a], Sarah J. Day [c], Lucy K. Saunders[c], Duncan N. Johnstone[b], Paul A. Midgley[a] *

[a] Department of Materials Science and Metallurgy
University of Cambridge
27 Charles Babbage Road, Cambridge, CB3 0FS, United Kingdom

[b] GSK R&D
Gunnels Wood Road, Stevenage, SG1 2NY, United Kingdom

[c] Diamond Light Source Ltd, Beamline I11
Harwell, Oxford, United Kingdom

*corresponding authors: hl585@cam.ac.uk pam33@cam.ac.uk

Supplementary Note 1. Sample Preparation of Xanthine Crystals for 3D-Electron Diffraction and 3D-Electron Diffraction Data Processing

Initially, xanthine powder (purchased from Sigma Aldrich, X7375, batch WXBD7599V) was directly deposited on Quantifoil grids in procedures described in previous work[1]. Attempts to grow sufficiently large single crystals from solution were unsuccessful and resulted in polycrystalline spherulitic aggregates. An aqueous suspension of xanthine powder was made using 0.5 mg of xanthine in 14.5 ml of distilled water. 5 μL of this suspension was micro-pipetted and dropped directly onto Quantifoil grids. Grids were left in a fume cupboard to allow the water to evaporate under room temperature conditions. This left behind more evenly distributed xanthine crystals sufficiently isolated for 3D-ED.

3D-ED data from particles which were single crystals were selected for further analysis. For these datasets, the tilt series was used to reconstruct reciprocal space, indexed, and integrated using CrysAlisPro 1.171.43.110a (Rigaku Oxford Diffraction, 2024)[2].

Supplementary Note 2. Structure Solution and Refinement from Twinned Dataset-Form I

The presence of two differently oriented lattices with low reflection overlap was seen in one particle (Figure S2.1). Unit cell dimensions for the two lattices were consistent with Form I xanthine[3], as shown in Table S2.1.

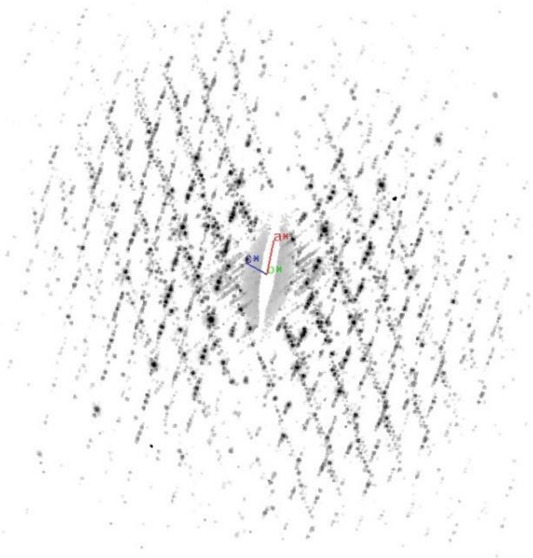

Figure S2.1 A view of the reconstructed 3D reciprocal space where the presence of two differently oriented lattices with low reflection overlap can be seen.

	a (Å)	*b* (Å)	*c* (Å)	*α* (°)	*β* (°)	*γ* (°)	*Volume* (Å^3)
**Form I**[1]	9.82(11)	17.87(8)	6.79(13)	90.0	107.5(9)	90.0	1136(26)
**Form I: first lattice in twinned crystal**	9.82(9)	17.86(13)	6.80(11)	90.0	107.8(7)	90.0	1136(20)
**Form I: second lattice in twinned crystal**	9.84(13)	17.89(14)	6.81(12)	90.0	107.7(9)	90.0	1142(20)

Table S2.1 Lattice parameters of Form I xanthine from separate experimental sessions are compared, showing that both of the lattices present in the particle are consistent with Form I xanthine.

Supplementary Note 3. Identifying Twins in 3D-ED Data – Form I

We considered two possibilities for the overlaid presence of two lattices observed in reconstructed reciprocal space: (1) these particles could consist of two overlaid but separate crystals with no crystallographic relationship to each other; (2) the particle could be a twinned crystal. In either of these cases, diffraction from both domains within the particle are superposed in the 3D-ED dataset. Along any common twinning plane, there are overlapping reflections from the planes common to both domains. In a non-merohedral twin (as is the case here) reciprocal lattices of twin components do not directly overlap and are more obviously observed upon initial data reduction[4]. A lack of perfectly overlapped reflections allows integration of indexed reflections corresponding to each individual lattice. Intensities remain relatively undisturbed which could otherwise hamper attempts at structure solution[5] such as in pseudo or merohedric twinning, when reflections may be indistinguishable because they fall on top of each other. The orientation matrices of the two observed diffraction patterns have a symmetric relationship. This means that the same transformation matrix can be applied to get from one to another. The 2-fold orientation relationship between both lattices shows that the two domains are related by either a 180 ° rotation or reflection. Figure S3.1 describes the twinning mechanism observed here in xanthine, with ±($10\bar{1}$) planes remaining invariant and unchanged by the effect of the twin, defined as the composition surface. This is also the surface along which lattice points in the twin are shared. We note that any 2-fold twin rotation axis (with the direction [1v1], where v can take any value such that the rotation axis lies within the ($10\bar{1}$) hydrogen-bonded planes) could in principle lead to alternative twinning which would still act to preserve the hydrogen-bonded planes in xanthine.

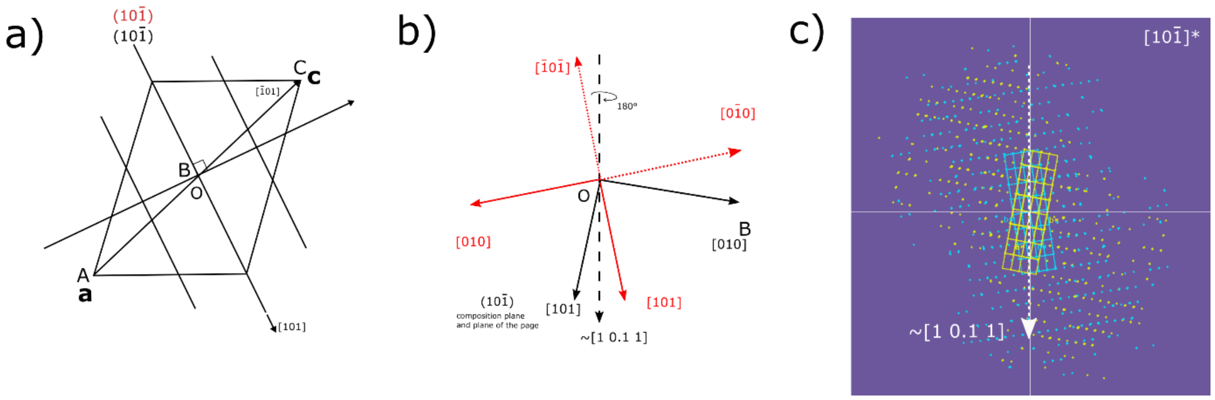

Figure S3.1 Supporting figure to Figure 2 in the main text (a is a repeat of Figure 2c) for ease of reference. The twinning presented here can be described using a 180° rotation axis about ~ [1 0.1 1], shown by the dashed black line in b). b) a schematic showing the action of the twin between the two lattices (with vectors shown in black and red). The plane of the paper is the (10$\bar{1}$) plane. c) a projection of the reciprocal space data parallel to [10$\bar{1}$]* (nearly parallel to [10$\bar{3}$]) to help visualise the schematic in b). This shows the twinning relationship (a 180° rotation around the [1 0.1 1] direction, which lies in the (10$\bar{1}$) plane). The relationship can also be described as a rotation about the [10$\bar{1}$]* axis of 19°. In c), one lattice is shown in blue, the other shown in yellow.

Supplementary Note 4. Form II xanthine polymorph structure solution

A limited angular range data collection (210 frames covering 105°) led to a low completeness of 79%. As a result, there was insufficient information regarding systematic absences to unambiguously identify the space group from reciprocal sections alone, which indicate a primitive orthorhombic lattice (Table S4.1). Regardless, attempts were made to deduce the likely space group. From the (0*kl*) section, (Figure 3a), reflections appear to obey an $0k0 = 4n$ symmetry rule. Given the primitive Bravais lattice, the origin of the missing reflections must instead originate from atomic positions in the polymorph such that the relevant structure factors are zero leading to missing reflections.

From the (*hk*0) and (0*kl*) sections, it was possible to identify the presence of two 2_1 symmetry operators. It was not possible to make this observation in the (*h*0*l*) slice due to the size of the missing wedge. As a result, $P2_12_12$ or $P2_12_12_1$ space groups were both consistent with the symmetric absences experimentally observed. $P2_12_12_1$ is a common space group, found in 18% of all CSD small molecule structures. On this basis, we attempted structure solution in the $P2_12_12_1$ space group. As was found with previously twinned particles, it was possible to integrate lattice intensities despite the particle being a bi-crystal because the majority of reflections from the two domains did not overlap (1% of indexed reflections overlapped as reported by CrysAlisPro).

In addition to two crystal domains and low completeness of data, a further challenge to the structure solution was the complexity of the asymmetric unit resulting from the higher number of independent xanthine molecules (Z' = 4) in the $P2_12_12_1$ space group, double that compared to the monoclinic $P2_1/c$ phase (Z' =2). Structure solution was successful in $P2_12_12_1$ using Dual Space methods implemented in SHELXD and was followed by kinematical refinement using SHELXL. Atoms were labelled as shown in Figure S4.1.

	a (Å)	*b* (Å)	*c* (Å)	*α* (°)	*β* (°)	*γ* (°)	*Volume* (Å^3)
**Form I: monoclinic**	9.82(11)	17.87(8)	6.79(13)	90.0	107.5(9)	90.0	1136(26)
**Form II: orthorhombic**	10.10(10)	12.54(10)	17.91(17)	90.0	90.0	90.0	2269(36)

Table S4.1 Comparison between Form I and Form II lattice parameters.

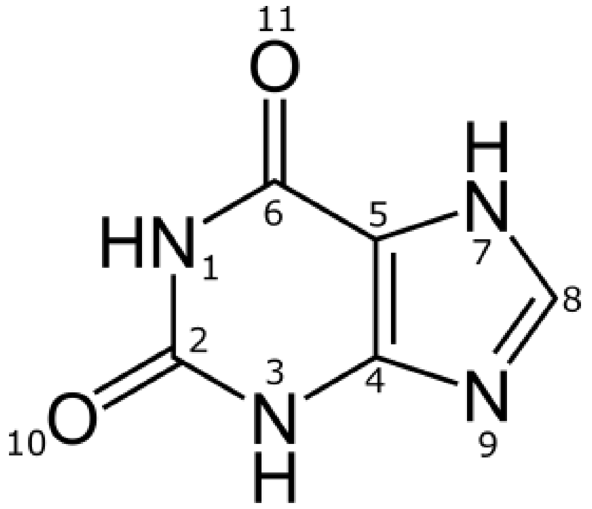

Figure S4.1 Labels used for structure refinement of xanthine. The three remaining xanthine molecules in the asymmetric unit have analogous atomic labelling, starting from N21 for the 2nd molecule, N41 for the 3rd, and N61 for the 4th.

Identification code	xanthine_form_II	
Empirical formula	C5 H4 N4 O2	
Formula weight	152.11	
Temperature	87(2) K	
Wavelength	0.0197 Å	
Crystal system	Orthorhombic	
Space group	P 21 21 21	
Unit cell dimensions	a = 10.10(10) Å	α= 90°.
	b = 12.54(10) Å	β= 90°.
	c = 17.91(17) Å	γ = 90°.
Volume	2269(36) $Å^3$	
Z	16	
Density (calculated)	1.781 Mg/m^3	
Absorption coefficient	0.000 mm^{-1}	
F(000)*	433	
Crystal size	0.001 x 0.001 x 0.0001 mm^3	
Theta range for data collection	0.055 to 0.627°.	
Index ranges	-11<=h<=11, -13<=k<=13, -18<=l<=18	
Reflections collected	6919	
Independent reflections	2605 [R(int) = 0.2653]	
Completeness to theta = 0.627°	79.4 %	
Refinement method	Full-matrix least-squares on F^2	
Data / restraints / parameters	2605 / 206 / 145	
Goodness-of-fit on F^2	1.230	
Final R indices [I>2sigma(I)]	R1 = 0.1706, wR2 = 0.4167	
R indices (all data)	R1 = 0.2799, wR2 = 0.4985	
Extinction coefficient	526(86)	
Largest diff. peak and hole	0.218 and -0.193 e.$Å^{-3}$ †	

Table S4.2 Selected parameters from the structure refinement using SHELXL, generated by XCIF. The structure solution and refinement executed here makes use of workflows which come from X-ray crystallography protocols. As such, we recognise that some parameters, such as the semi-empirical absorption corrections which may account in practice for effects of beam damage, are not ideal for use with electron diffraction. *This number represents the sum of the electron structure factors at a zero scattering angle. † These units which result from SHELXL refer to X-ray scattering factors. However, electron scattering factors are used here, so the units that are correct should be $Å^{-2}$.

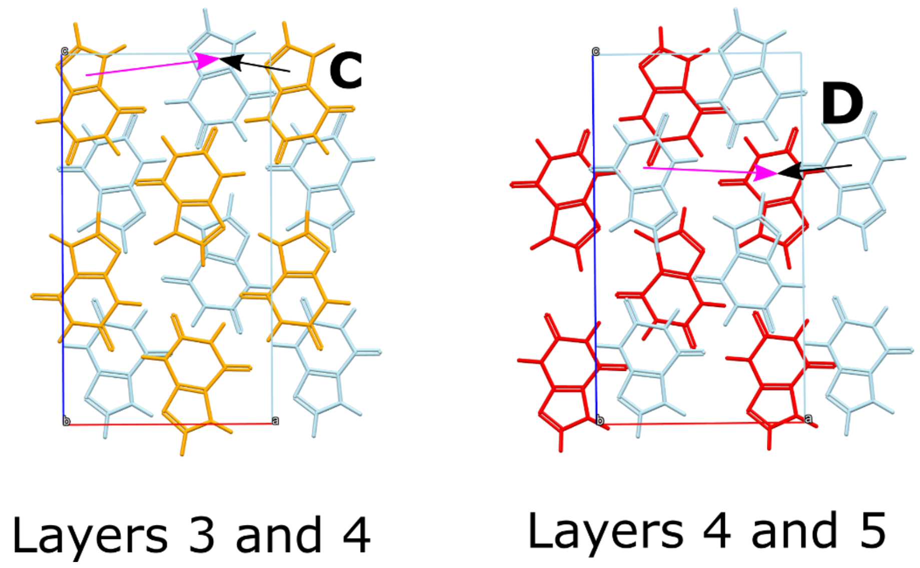

Figure S4.2 A supplementary figure to support Figure 5 in the main text. The pink arrow shows an alternative way to define the interlayer vectors in Form II Xanthine, keeping a consistent direction with vectors **A** and **B** as shown in Figure 5. However, we choose to define the interlayer vector using the black arrow to simplify the number of unique vectors present and to demonstrate the relation of **A, B, C,** and **D**.

Supplementary Note 5. 4D-STEM: another example particle

The variable extent of streaking across different summed patterns demonstrates that planar disorder is not homogenous across all domains of the particle. Another example of this observation from another particle is shown in Fig S5.1. The summed diffraction pattern in S5.1b) is indexable with no signs of streaking, the pattern in S5.1c) has no Bragg spots except those corresponding to the stacked layers (although this could also be partly due to the signal coming from a thicker part of the particle, and the dominant effects of inelastic scattering), and the pattern in S5.1d) has prevalent streaked Bragg reflections (most easily seen in th strong systematic rows) consistent with displacements of the layers. VDFs formed by placing apertures over reflections corresponding to the layered stacking would be expected to show even contrast across the particle. A virtual aperture placed over one of these reflections (the aperture shown in Fig S5.1c) shows interesting contrast in the VDF, Fig S5.1f). As expected, contrast is not constrained to one domain. However, the edges of the particle show higher contrast than the center. This likely results from the greater thickness at the center of the particle. This is supported by mass-thickness contrast in the High Angle Annular Dark Field (HAADF) image (Fig S5.1a).

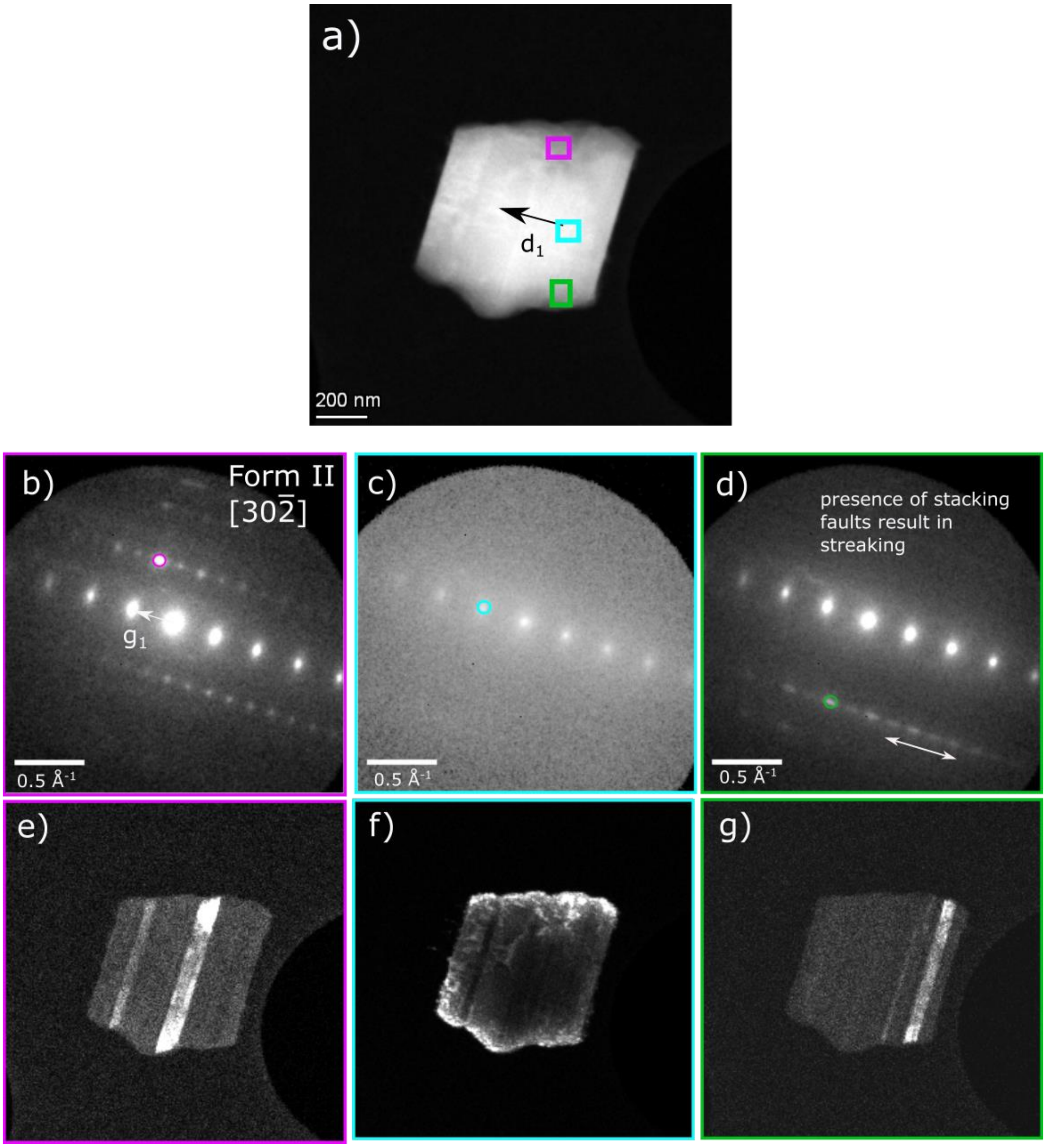

Figure S5.1 a) High Angle Annular Dark Field (HAADF) image of a xanthine crystal. Diffraction signal from the areas highlighted in different colours are used to form the summed diffraction patterns shown in b-d). e-g) Virtual Dark Field (VDF) images are formed from apertures circled in these summed patterns and are highlighted in corresponding colours. VDF contrast indicates the presence of slab-like domains within the crystals with interfaces parallel to the stacked layers. f) VDF corresponding to the stacking vector, which should remain relatively consistent throughout the entire particle. We postulate that the edges are highlighted likely due to the thicker center of the particle.

Supplementary Note 6. X-ray Powder Diffraction Studies

TOPAS academic software was used to perform Rietveld refinements on XRPD data[7] collected as described in the Methods section. In previous work[3], modelling only Form I xanthine to this data refined to R_{wp} = 5.97% and GoF = 8.27%.

A multiphase Rietveld refinement including both Form I and Form II led to the improved fit as seen in Figure S6.1 (R_{wp} = 2.36%, GoF = 3.72%). The measurement of an empty capillary was used as an empirical background with a scale factor which was set as a refinable parameter.

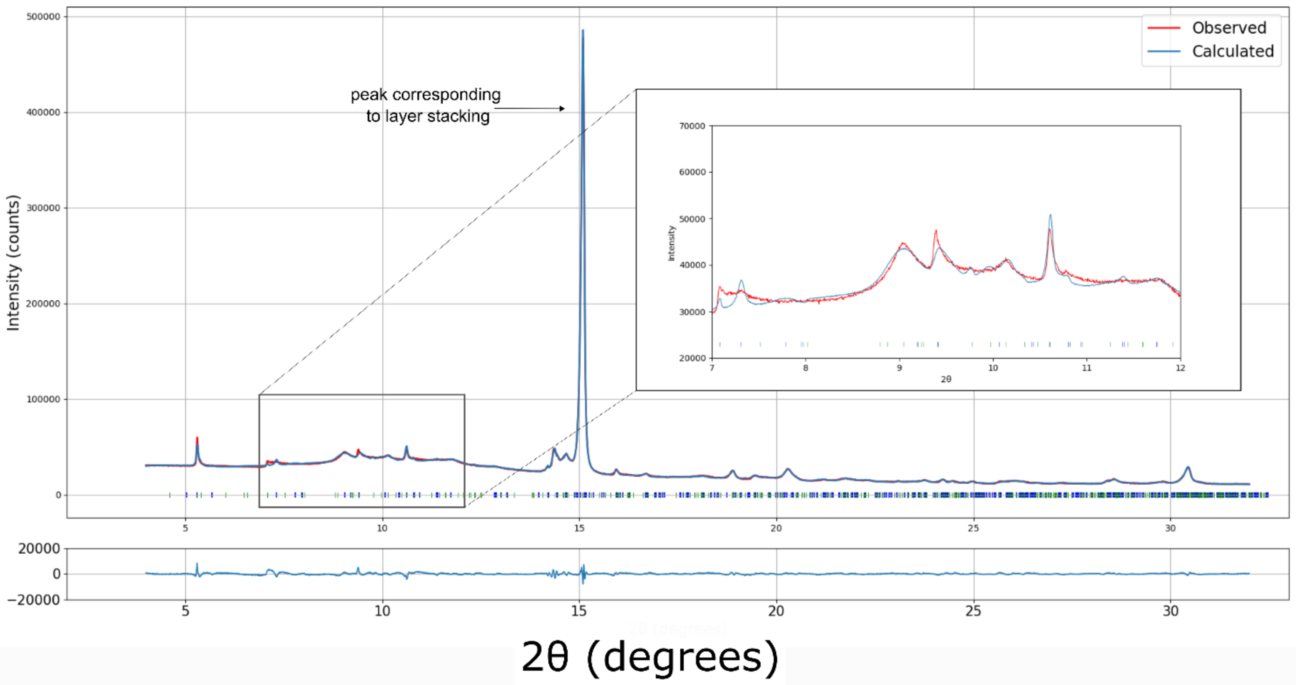

Figure S6.1 A multiphase Rietveld refinement of XRPD data including both Form I and Form II xanthine structures. This led to a fit of R_{wp} 2.36% and Goodness of Fit of 3.72%. The inset shows an improved fit compared to a single-phase refinement, with all peaks now accounted for. However, the fit was further improved by the inclusion of stacking faults in the Rietveld model (see main text). Expected peak positions for the two phases are shown by the ticks in blue (Form I), green (Form II).

To improve the model, stacking faults were introduced. A supercell based on the orthorhombic geometry of Form II was modelled, for simplicity consisting of 20 layers (5 stacked unit cells of Form II parallel to the b-axis). Insights from 3D-ED structural data (shown in Figure 5b) were used to generate stacking faults randomly distributed throughout the supercell. The starting position of each of the 20 layers followed a perfect **ABCD** packing (specifically; **DABCDABCDABCDABCDAB**). Stacking faults were introduced at random layers in the supercell, and were restrained to either **A**, **B**, **C**, or **D** vectors whilst the atomic arrangements within each layer were fixed. 100 different starting arrangements of stacking fault sequences were randomly generated and refined. Form I was also included in these refinements but without modelling stacking faults due to higher model complexity using the supercell approach. This can be justified given that the stacking sequence of Form I is a subset of possible vectors found in Form II. For each starting arrangement, the R_{wp} was noted. The starting model with the lowest R_{wp} was used to run final refinements (Figure 9, R_{wp} = 1.55% and GooF = 2.46%). The range of R_{wp} values for all the generated models was narrow: 1.55% to 2.34%. In our models, we allowed for some minimal rigid body refinement, but the height between each layer

remained fixed. The final supercell stacking sequence resulting in the lowest R_{wp} was: **D*BCD*DABCDABC*B*ABCDAB**. The ‘out of sequence’ vectors have been italicised. To support this model, simulated electron diffraction patterns using the model with irregularly distributed stacking faults have streaking broadly consistent with experimentally observed electron diffraction data (Figure S6.2).

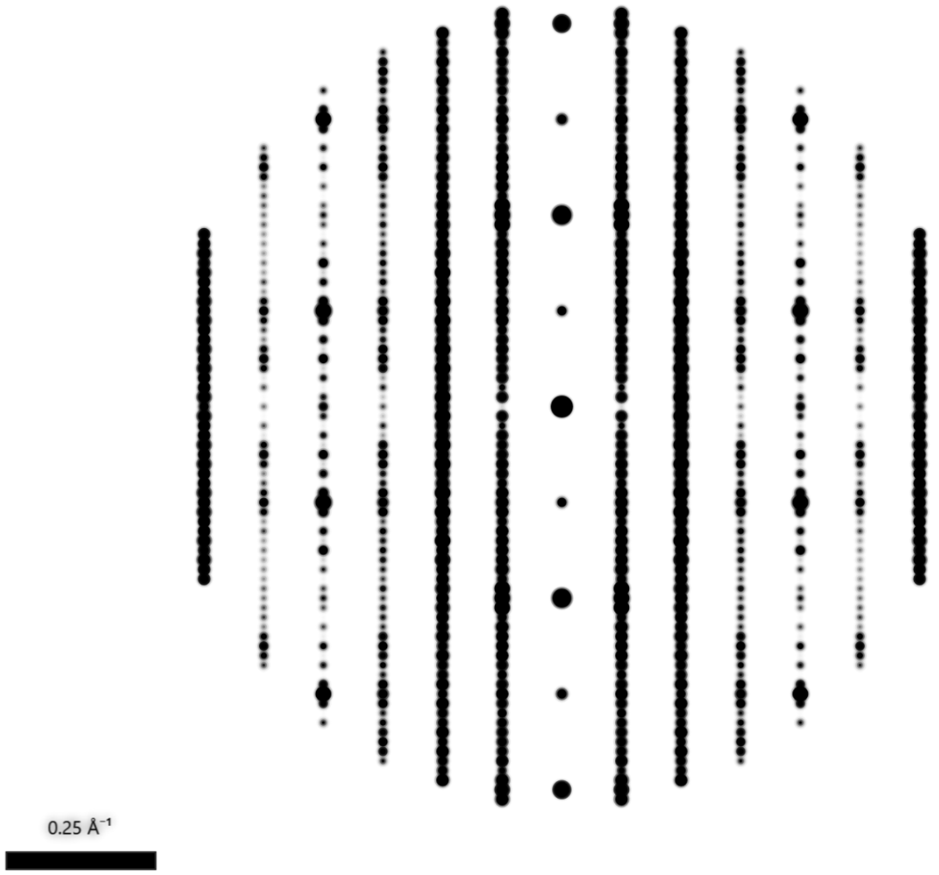

Figure S6.2 Simulated electron diffraction pattern [001] of the stacking fault model. This model consists of a supercell of 20 layers based on Form II xanthine. Stacking faults were introduced at random into the starting model based on structural insights from 3D-ED data. The streaking effect seen above is consistent with observations in electron diffraction data.

Supplementary Note 7. Comparison of Xanthine with other Planar Organic Molecules

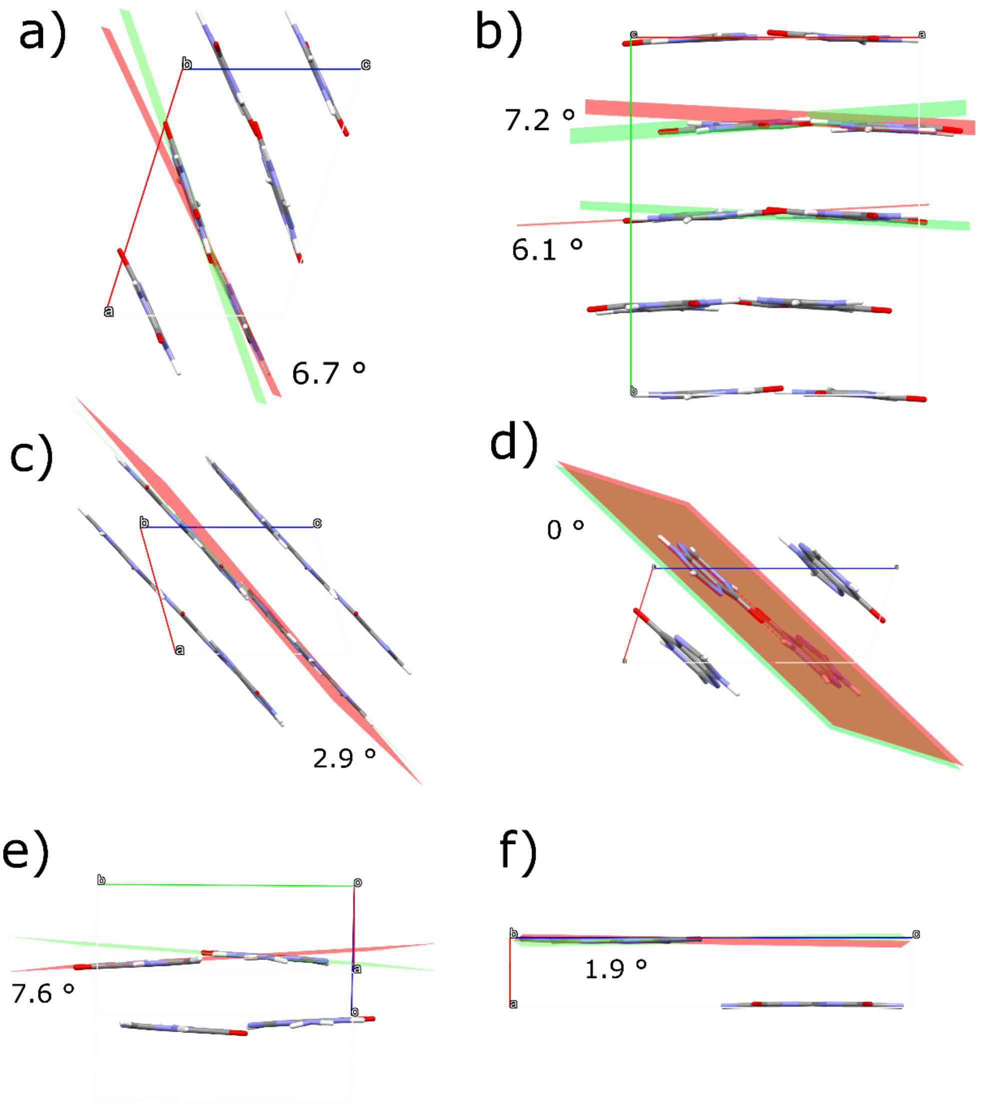

Figure S7.1 Side-on view of planar structures with mean planes drawn through molecules pictured in red and green. The angle between the red and green planes is shown for: (a) xanthine Form I (b) xanthine Form II (c) hypoxanthine triclinic form (d) hypoxanthine monoclinic form (e) α guanine (f) β guanine.

As illustrated in Figure S7.1b), the out-of-plane tilt of xanthine molecules varies in Form II xanthine, with molecules in one layer canted at an angle of 7.2 ° and the other at 6.1 °. In comparison, all molecules in the layers of Form I xanthine have an identical tilt of 6.7 °. Whilst the energy differences between these positions are likely very similar, this small angular variation might lead to favoured stacking sequences in Form II, with alternate layers preferring to 'switch' positions: i.e. that the action of a stacking fault might favour moving layer 1 into the position of layer 3 to keep the inter-layer angles consistent (and layer 2 into a layer 4 position). Extending this possibility, it is interesting to note that a consistently preferred stacking sequence change in which layers 1 and 3 and layers 2 and 4 were swapped would lead to microtwins: **ABCDABCDAB|ADCBADCBAD** (where | denotes the twin boundary/stacking fault).

Supplementary References

1. Leung, H. W. *et al.* Revealing the Crystal Structure of the Purine Base Xanthine with Three-Dimensional (3D) Electron Diffraction. *Cryst. Growth Des.* (2025) doi:doi/10.1021/acs.cgd.4c01594.

2. Rigaku Oxford Diffraction. CrysAlisPro 1.171.43.110a. (2024).

3. Leung, H. W. *et al.* Revealing the Crystal Structure of the Purine Base Xanthine with 3D Electron Diffraction. *ChemRxiv* (2024) doi:10.26434/CHEMRXIV-2024-TLGVB.

4. Sevvana, M., Ruf, M., Uson, I., Sheldrick, G. M. & Herbst-Irmer, R. Non-merohedral twinning: From minerals to proteins Sevvana Madhumati. *Acta Crystallogr. Sect. D Struct. Biol.* **75**, 1040–1050 (2019).

5. Carri, M. & Ferraris, G. Twinning by Merohedry and X-ray Crystal Structure Determination. *Acta Cryst* **32**, 163 (1976).

6. Il B Y, B. A. B., Crookerf, A. G. & Cottrell, A. H. The theory of the crystallography of deformation twinning. *Proc. R. Soc. London. Ser. A. Math. Phys. Sci.* **288**, 240–255 (1965).

7. A. Coelho. TOPAS-Academic V5 . (2012).
