# Supplementary material for: Polytypes and planar defects revealed in the purine base xanthine using multi-dimensional electron diffraction
Source: Commun Chem. 2025 Nov 5;8:331. doi: 10.1038/s42004-025-01729-2 (PMC12589599; doi:10.1038/s42004-025-01729-2)
Supplement: Supplementary file 3 — Description of Additional Supplementary Files [file 42004_2025_1729_MOESM3_ESM.pdf]

Description of Additional Supplementary Files

File name: Supplementary Data 1

Description: Crystallographic Information File for xanthine Form II. Referred to as 'Supplementary Data 1' in the data availability statement in the main manuscript.

File name: Supplementary Data 2

Description: Input file for TOPAS used to perform Rietveld refinement. This model uses Form I and Form II xanthine crystal structures. Referred to as 'Supplementary Data 2' in the data availability statement in the main manuscript.

File name: Supplementary Data 3

Description: Input file for TOPAS used to perform Rietveld refinement. This model uses Form I and Form II xanthine crystal structures in addition to a supercell stacking faults model. Referred to as 'Supplementary Data 3' in the data availability statement in the main manuscript.
